# Early increase in HbA1c trajectory predicts development of severe microangiopathy in patients with type 1 diabetes: the VISS study

**DOI:** 10.1136/bmjdrc-2023-003917

**Published:** 2024-05-06

**Authors:** Hans J Arnqvist, Johnny Ludvigsson, Maria Nordwall

**Affiliations:** 1 Division of Endocrinology, Department of Biomedical and Clinical Sciences, Linköping University, Linkoping, Sweden; 2 Division of Pediatrics, Department of Biomedical and Clinical Sciences, Linköping University, Linkoping, Sweden; 3 Crown Princess Victoria Childrens Hospital, Linköping, Sweden; 4 Division of Pediatrics, Vrinnevi Hospital in Norrköping, Norrkoping, Sweden

**Keywords:** Diabetes Mellitus, Type 1, Diabetic Angiopathies, Glycated Hemoglobin A

## Abstract

**Introduction:**

To study the HbA1c trajectory from the time of diagnosis to examine if patients at the greatest risk for severe microangiopathy can be identified early allowing clinicians to intervene as soon as possible to avoid complications.

**Research design and methods:**

In a population-based observational study, 447 patients diagnosed with type 1 diabetes before 35 years of age, 1983–1987, were followed from diagnosis until 2019. Mean HbA1c was calculated each year for each patient. Severe diabetic microangiopathy was defined as proliferative diabetic retinopathy (PDR) or macroalbuminuria (nephropathy).

**Results:**

After 32 years, 27% had developed PDR and 8% macroalbuminuria. Patients with weighted HbA1c (wHbA1c); <57 mmol/mol; <7.4% did not develop PDR or macroalbuminuria. The HbA1c trajectories for patients developing PDR and macroalbuminuria follow separate courses early on and stay separated for 32 years during the follow-up. Patients without severe complications show an initial dip, after which HbA1c slowly increases. HbA1c in patients with severe complications directly rises to a high level within a few years. Mean HbA1c calculated for the period 5–8 years after diabetes onset strongly predicts the development of severe complications. Females with childhood-onset diabetes exhibit a high peak in HbA1c during adolescence associated with higher wHbA1c and higher prevalence of PDR.

**Conclusions:**

The HbA1c trajectory from diabetes onset shows that mean HbA1c for the period 5–8 years after diagnosis strongly predicts severe microangiopathy. Females with childhood-onset diabetes exhibit a high peak in HbA1c during adolescence associated with higher wHbA1c and a higher prevalence of PDR.

**WHAT IS ALREADY KNOWN ON THIS TOPIC**

- The development of diabetic retinopathy and nephropathy in patients with type 1 diabetes is related to diabetes duration and hyperglycemia assessed as HbA1c.

**WHAT THIS STUDY ADDS**

- An early increase in HbA1c trajectory strongly predicts the development of proliferative diabetic retinopathy and diabetic nephropathy. Females with childhood-onset diabetes exhibit a high peak in HbA1c trajectory during adolescence associated with higher long-term mean weighted HbA1c (wHbA1c) and higher prevalence of PDR.

**HOW THIS STUDY MIGHT AFFECT RESEARCH, PRACTICE OR POLICY**

- To prevent severe microangiopathy, improved diabetes care directed to early intervention in patients with a steep rise in HbA1c and in adolescent girls is needed.

## Introduction

 Glycosylated hemoglobin was characterized in the late 1970s and introduced as a biomarker of average glycemia in the early 1980s.^1^ Using this new tool, the landmark study Diabetes Control and Complications Trial (DCCT) convincingly showed that diabetic microangiopathy and macroangiopathy in type 1 diabetes are related to the level of hyperglycemia.^2^ Despite the data from DCCT, achieving targeted glycemia has remained elusive for the majority living with type 1 diabetes. International benchmarking in type 1 diabetes shows large differences in HbA1c between high-income countries.^3^ Several studies have focused on HbA1c trajectories during adolescence and early adulthood.^4^,^6^ Despite the different HbA1c levels, similarities in HbA1c trajectories occur.^4^ Understanding the trajectory of HbA1c after diagnosis can target interventions in the course of type 1 diabetes.^7^ In a register study, HbA1c level during childhood has been shown to have an association with glycemic control in adulthood and the risk of diabetic retinopathy.^8^ In Sweden, teenage girls with type 1 diabetes have poorer metabolic control than boys and get more complications in early adulthood.^9^

In a long-term follow-up, we studied patients with type 1 diabetes aged 0.5–34 years at diagnosis with HbA1c from diabetes onset and showed that the long-term mean weighted HbA1c (wHbA1c) calculated by integrating the area under all HbA1c values from diagnosis and dividing by time of follow-up is a very strong predictor of severe retinopathy and nephropathy.^10^

Our aim now is to study the HbA1c trajectory from the time of diagnosis when HbA1c is expressed as yearly mean values to examine if patients at the greatest risk for severe microangiopathy can be identified early allowing clinicians to intervene as soon as possible to avoid complications.

## Materials and methods

### Patients

All 447 patients with onset of type 1 diabetes before the age of 35 years, January 1, 1983, to December 31, 1987, in the Southeast hospital region in Sweden were studied. That all patients were included was validated with the Swedish Childhood Diabetes Registry^11^ and the Diabetes Incidence Study in Sweden (DISS).^12^ A follow-up of the original patient cohort was made in January 2019, that is, at a diabetes duration of 32–36 years. Data were collected from medical records as previously described.^10^ Data on 432 of the 447 patients could be retrieved from the Swedish National Diabetes Registry (NDR).^13^ 14 patients had their last clinical visit before the start of NDR 1996 and 1 patient had not been registered. Information was also retrieved from the Swedish Renal Registry,^14^ the Swedish National Patient Register and the Swedish Cause of Death Register. Patients who had moved abroad (n=7) were followed to their last visit in Sweden and patients who were deceased (n=55) were followed to their last visit.

### Insulin treatment

In the 1980s, almost all newly diagnosed patients in Sweden used intensive insulin regimens from the beginning. During the last decades, insulin analogs have been introduced and the number of patients using sensor-based glucose testing, continuous glucose monitoring (CGM), has increased. When the data was retrieved from NDR, 62% of the patients used CGM and 23% used insulin pumps.

### Retinopathy

The retinal screening was done using fundus photography with one central and one nasal field per eye. The level of retinopathy and macular edema was classified according to the International Clinical Diabetic Retinopathy and Diabetic Macular Edema Disease Severity Scale.^15^ The indication for treatment with laser or intraocular injections was either proliferative diabetic retinopathy (PDR) or diabetic macular edema. The date of the first treatment was collected from clinical records. The prevalence of PDR was stated at the end of the study. Photographs or reliable data concerning previous therapy with laser or injections for PDR and/or maculopathy were available for 440 (98%) of the 447 patients.

### Nephropathy

The patients were screened for proteinuria at their regular clinical visits, at least once every year. The urine sample was analyzed with quantitative immunoturbidometric methods. Macroalbuminuria was defined as persistent albumin excretion rate (AER)>200 µg/minute or albumin/creatinine ratio >30 mg/mmol. For all patients with macroalbuminuria, the medical records were scrutinized to confirm that there was no other kidney disease than diabetic nephropathy explaining the condition. Data were available for 441 (99%) of the patients. One patient had IgA nephropathy and one patient with myelomeningocele had hydronephrosis and they were excluded leaving 439 patients for renal evaluation. At the follow-up, 45% of the patients were treated with antihypertensive drugs mainly blockers of the renin–angiotensin–aldosterone system. The prevalence of macroalbuminuria was stated at the end of the study.

### HbA1c measurement

HbA1c was measured regularly, two to four times per year. The HbA1c methods have been described previously.^10^ In 1997, a nationwide standardization was introduced in Sweden with a standardization scheme based on the Mono S method.^16^ Repeated comparisons were made with National Glycohemoglobin Standardization Program (NGSP) values, which showed the Swedish values to be 1.1% lower than NGSP values.^17^ The same was demonstrated in a study comparing HbA1c measured in 1994 at the Linkoping University Hospital Laboratory with the DCCT laboratory.^18^ Since 2007, the HbA1c method has been internationally standardized.^19^ All values in this report are converted by formulas to the new International Federation of Clinical Chemistry and Laboratory Medicine (IFCC) reference values. The corresponding NGSP values are also stated, making it possible to compare the results with previous studies. The normal range is 27–42 mmol/mol (IFCC) corresponding to 4.6–6.0% (NGSP).

90% of the HbA1c values came from laboratories in the catchment area and were from 2004 collected directly from the central laboratories databases. In total, 36 550 HbA1c values were collected, 82±27 (mean±SD) values per patient and on average 2.6±0.6 values per patient and year. We checked for gaps in the HbA1c series by making a pivot table. Gaps of up to 3 years were accepted. Six patients with complications had gaps in the HbA1c series between 4 and 9 years before the complication: one with PDR alone, four with both PDR and macroalbuminuria and one with macroalbuminuria alone. Since the values before and after the gaps were very consistent, we estimated that the missing data would not affect the results. Nine patients with HbA1c gaps 10 years or longer were not included in the analysis of the impact of HbA1c on complications.

As a measure of long-term glycemic control, long-term wHbA1c was calculated by integrating the area under all HbA1c values from diagnosis and dividing by time.^10^ This results in one wHbA1c per patient. For evaluation of HbA1c trajectories, the analysis is based on individual yearly mean values.

### Statistical analysis

Data are shown as mean±SD unless otherwise stated. The significance level was set at p<0.05. SPSS V.28 was used for the analyses. To test the predictability of early HbA1c values on the development of PDR and macroalbuminuria, mean HbA1c during the period 5–8 years after diagnosis, when the HbA1c peak was reached, was calculated. The association with the prevalence of severe complications at the end of the study was then tested using quartiles. Differences between groups were tested with t-test or analysis of variance with Bonferroni post hoc test. The χ^2^ test was used for categorical variables.

## Results

### HbA1c trajectory—age, duration and sex

**Figure 1:**
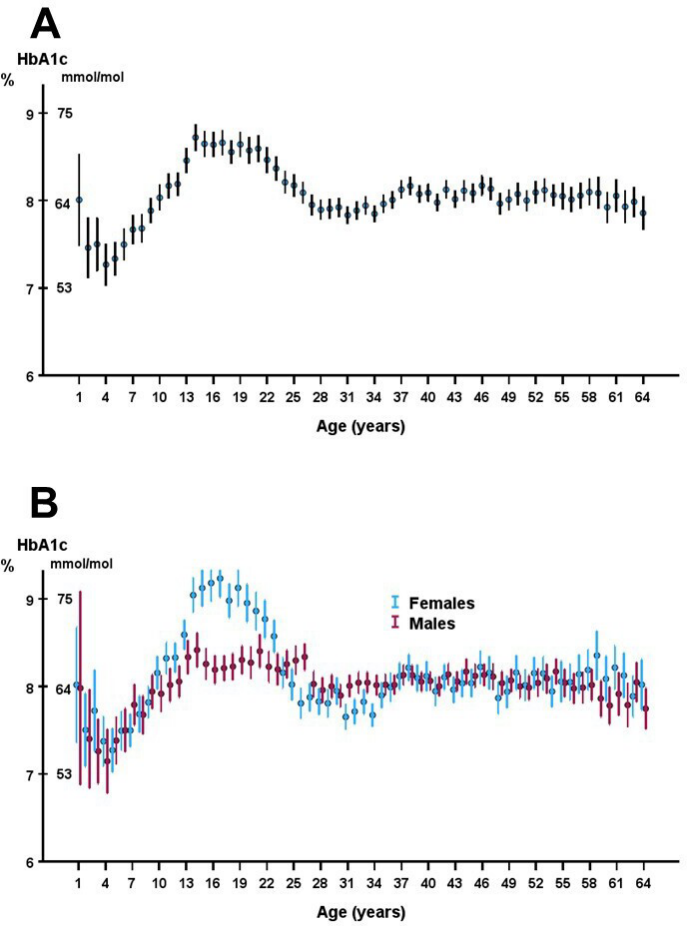
Yearly mean HbA1c values (**A**) in relation to age and (**B**) in relation to age and sex. Error bars denotes 95% CIs.

**Figure 2:**
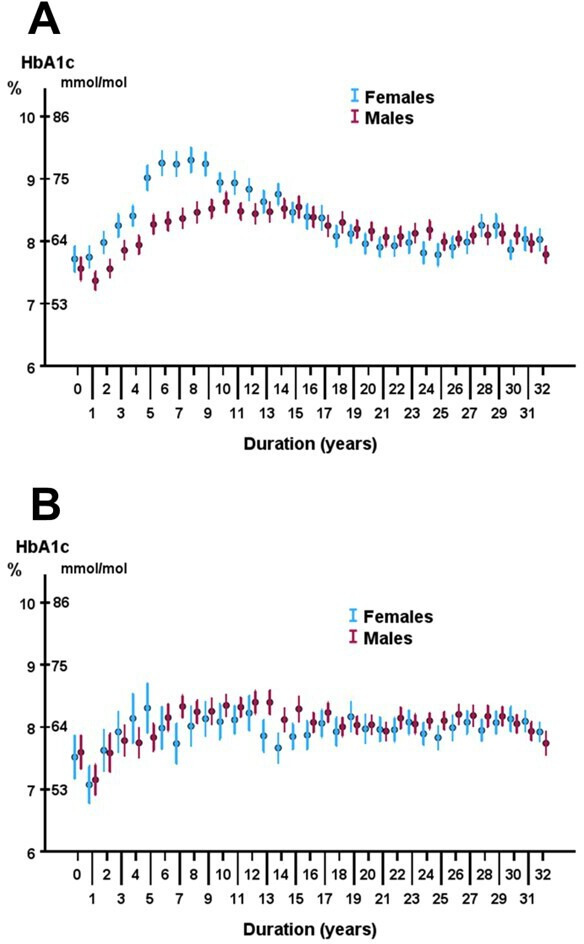
Yearly mean HbA1c values in patients with type 1 diabetes, (**A**) in relation to diabetes duration in males and females diagnosed before 18 years of age and (**B**) in relation to diabetes duration in males and females diagnosed at an age of 18 years or later. Error bars denotes 95% CIs.

The trajectory for yearly mean HbA1c values with age showed initially low values with a rise to a hump starting at 15 years and leveling off at 22 years (figure 1A). When making separate graphs for males and females, it is obvious that the hump is mainly due to high HbA1c in adolescent females and young adult women (figure 1B). In figure 2A, mean HbA1c for males and females is shown according to diabetes duration for patients with diabetes onset before 18 years of age. After 5 years’ duration, the difference between males 65±18 mmol/mol; 8.1%±1.7% and females 73±19 mmol/mol; 8.8%±1.7% was significant, p<0.001. The largest difference was found after a duration of 8 years, with males 68±18 mmol/mol; 8.4%±1.7% and females 78±19 mmol/mol; 9.3%±1.7%, p<0.001. In patients diagnosed at an age of 18 years or older, there was no sex difference in HbA1c trajectories and for females, no peak was seen after a diabetes duration of 5 years (figure 2B).

### HbA1c trajectory—complications

The main population features divided by PDR and nephropathy status are given in table 1. After 32 years, 118 (27%) patients had developed PDR and 34 (8%) patients had developed macroalbuminuria. Sex distribution and age at onset did not differ significantly between patients with or without complications. Patients with PDR had higher wHbA1c values 74±10 mmol/mol; 8.9%±0.9% (mean±SD) in comparison with those without PDR wHbA1c 63±10 mmol/mol; 7.9%±0.9% (p<0.001). The minimum value for the development of PDR was wHbA1c 57 mmol/mol; 7.4%. Compared with patients with PDR, patients with nephropathy had higher wHbA1c, 82±12 mmol/mol; 9.7%±1.1% versus 74±10 mmol/mol; 8.9%±0.9% (p<0.001). The minimum wHbA1c value for a patient with macroalbuminuria was 64 mmol/mol; 8.0%.

**Table 1:** Population features divided by PDR and nephropathy status

Variable	No PDR	PDR	P value	No macroalbuminuria	Macroalbuminuria	P value
Individuals, n (%)	322 (73)	118 (27)	–	405 (92)	34 (8)	–
Women, n (%)	134 (42)	53 (45)	NS	175 (43)	13 (38)	NS
Debut age of diabetes (years)	16±9	15±9	NS	15±9	18±10	NS
wHbA1c (mmol/mol)	63±10	74±10	<0.001	64±10	82±12	<0.001
wHbA1c (%)	7.9±0.9	8.9±0.9	<0.001	8.0±0.9	9.7±1.1	<0.001

PDR, proliferative diabetic retinopathy.

**Figure 3:**
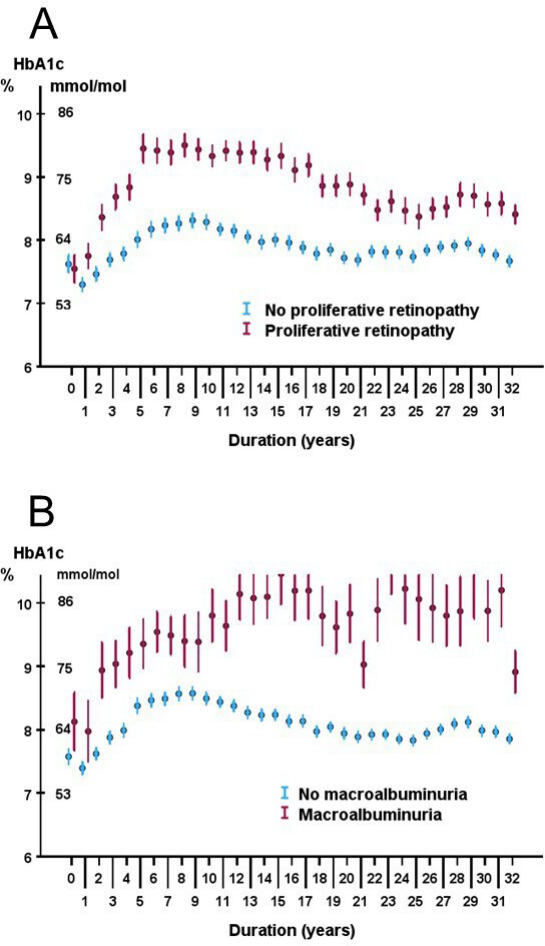
(**A**) Yearly mean HbA1c values in relation to duration in patients with and without proliferative diabetic retinopathy, (**B**) yearly mean HbA1c values in relation to duration in patients with and without macroalbuminuria. Error bars denotes 95% CIs.

Compared with patients without PDR, the HbA1c trajectory for patients developing PDR showed a much steeper rise during the first years and reached a peak after 5 years (figure 3A). In patients without PDR, there was an initial dip in the HbA1c curve and then a slow rise during the first 5 years. After 5 years’ duration, HbA1c in patients who later developed PDR was 75±19 mmol/mol; 9.0%±1.7% compared with 62±16 mmol/mol; 7.8%±1.5%, in those who did not develop PDR, p<0.001, illustrating the steep rise in the HbA1c trajectory. Up till the end of the observation period of 32 years, there was a clear separation in mean yearly HbA1c values including CIs. As shown in figure 3B, patients with nephropathy defined as macroalbuminuria had higher yearly mean HbA1c values almost from diabetes onset compared with patients without nephropathy.

**Figure 4:**
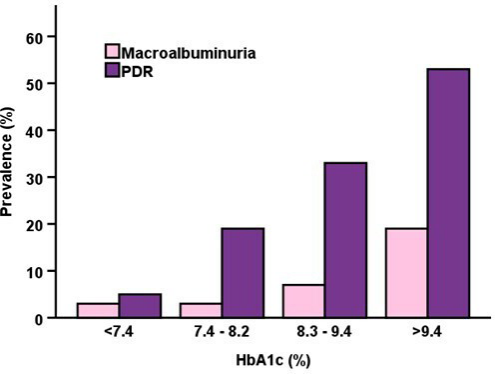
Quartiles of mean HbA1c for the period 5–8 years after diabetes onset related to PDR and nephropathy. Compared with HbA1c <7.4%, the increase was significant (p<0.001) at HbA1c 8.3–9.4% and >9.4% for PDR and >9.4% for macroalbuminuria (p<0.001). Expressed in mmol/mol <7.4%=<57; 7.4–8.2%=57–66; 8.3–9.4%=67–79; >9.4%=>79. PDR, proliferative diabetic retinopathy.

Mean HbA1c calculated for the period 5–8 years after diagnosis was strongly associated with the risk of developing PDR and nephropathy. As shown in figure 4, the prevalence of PDR in patients in the lowest quartile (HbA1c <57 mmol/mol; 7.4%) was only 4%, while in the highest quartile the prevalence was 47%. The prevalence of nephropathy was also related to HbA1c quartiles 5–8 years after diagnosis (figure 4).

During the follow-up period of 32 years, females diagnosed before 18 years of age who exhibited a high peak in HbA1c during adolescence had higher wHbA1c (67±11 mmol/mol; 8.3%±1%) than women 18 years or older at diagnosis (63±12 mmol/mol; 7.9%±1.1%, p=0.026). This was associated with a higher prevalence of PDR, 33% and 20%, respectively (p=0.038), while there was no significant difference for men. The prevalence of macroalbuminuria did not differ between women diagnosed before 18 years of age or later while for men the prevalence of macroalbuminuria was lower for those diagnosed before 18 years of age, 5% and 14%, respectively (p=0.018).

## Discussion

The HbA1c trajectory from diabetes onset was studied to see if patients with the greatest risk for severe microangiopathy can be identified early. Our main finding was that the HbA1c level when the trajectory peaks after a diabetes duration of 5–8 years strongly predicts the development of proliferative retinopathy and nephropathy during the follow-up time of 32 years. Compared with those who did not develop PDR, the trajectory for patients developing PDR followed a different path with higher HbA1c values throughout the 32 years. In a study from the NDR in Sweden, HbA1c levels during childhood were associated with HbA1c levels in adulthood and with retinopathy.^8^ HbA1c during the first year after diagnosis has been reported to be associated with retinopathy 25 years later.^20^ We tested to calculate mean HbA1c for the period 5–8 years after diagnosis when the maximum was reached and divided into quartiles (figure 4). Only 5 (4%) of 116 patients with complete HbA1c series who developed PDR during the follow-up of 32 years were found in quartile one with an upper HbA1c limit <57 mmol/mol; 7.4% compared with 55 patients (47%) in quartile 4. The risk for nephropathy also increased with higher quartiles but was only significant between quartiles 1 and 4. These observations suggest that patients at the greatest risk for the development of severe complications can be identified early, allowing clinicians to intervene promptly to avoid the onset of serious diabetic microangiopathy.

The prevalence of eye complications (PDR) is much higher than renal complications (macroalbuminuria), 27% vs 8%. This is in well agreement with the observation in the DCCT/EDIC study at 30 years with a prevalence of PDR of 31% and macroalbuminuria 8% in the conventional group.^21^ One interpretation of this is that a lower glycemic load is needed to precipitate eye complications than renal damage. In this study, the lowest wHbA1c for the development of PDR and macroalbuminuria were 57 mmol/mol; 7.4% and 64 mmol/mol; 8.0%, respectively.

The trajectory for the yearly mean values of HbA1c in relation to age increased to a peak at about 15 years of age. A rise in HbA1c during adolescence has been reported in several studies.^4^,^6^ Various factors may contribute to the deterioration in glycemic control such as hormonal changes in puberty, a decline in endogenous insulin secretion and psychological issues during puberty.^5 22 23^ Splitting the curve into males and females revealed that the peak was mainly due to high HbA1c values in female adolescents and young adults. In several but not all studies^5 9 24^ of glycemic control during adolescence, females are reported to have higher HbA1c values than males. In our study, it was only women with diabetes onset before 18 years of age who exhibited higher HbA1c values. This is in line with the report from Samuelsson *et al*^9^ who reported poorer metabolic control in teenage girls in comparison to boys. There can be several explanations for the findings that girls, to a higher extent than boys, have poor glycemic control during adolescence. Differences in insulin antagonistic hormones, insulin sensitivity, body mass index, decline of residual beta cell function, as well as psychological problems are factors considered to be of importance for these differences between sexes.^5 25 26^ It should be pointed out that in Sweden and many other countries, the patients are transferred from pediatric care to adult care at an age around 18, that is, at the same age as the HbA1c curve reaches its maximum in females.

Poor metabolic control in teenage girls was associated with an increased prevalence of diabetic retinopathy categorized as present or absent in another study in a Swedish population.^9^ In our study, the outcome was severe sight-threatening retinopathy, that is, PDR. The women with childhood-onset type 1 diabetes who exhibited a high HbA1c during adolescence also had a higher long-term wHbA1c compared with women with adult-onset diabetes and their prevalence of PDR was also higher. This argues for a need for improved pediatric diabetes care directed to adolescent girls to prevent complications.

Limitations of our study are that we can only speculate about the factors behind sex differences in HbA1c during adolescence due to lack of clinical data about treatment regimens, insulin sensitivity, endogenous insulin secretion and psychological issues. The strength is that the cohort included both children and adults, with a long and complete follow-up of HbA1c in the whole population. This enabled us to study the impact of the HbA1c trajectory on severe complications.

In conclusion, long-term follow-up of HbA1c from diabetes onset shows that the HbA1c trajectory during the first years is a strong predictor of severe microangiopathy. Females with childhood-onset diabetes exhibit a high peak in HbA1c during adolescence associated with higher wHbA1c and a higher prevalence of PDR.

## Data Availability

Data are available upon reasonable request.

## References

[1] Bunn HF, Gabbay KH, Gallop PM (1978). The glycosylation of hemoglobin: relevance to diabetes mellitus. Science.

[2] Nathan DM, Genuth S, Diabetes Control and Complications Trial Research Group (1993). The effect of intensive treatment of diabetes on the development and progression of long-term complications in insulin-dependent diabetes mellitus. N Engl J Med.

[3] Anderzén J, Hermann JM, Samuelsson U (2020). International Benchmarking in type 1 diabetes: large difference in childhood Hba1C between eight high-income countries but similar rise during adolescence-a quality registry study. Pediatr Diabetes.

[4] Sherr JL, Schwandt A, Phelan H (2021). Hemoglobin A1C patterns of youth with type 1 diabetes 10 years post diagnosis from 3 continents. Pediatrics.

[5] Bryden KS, Peveler RC, Stein A (2001). Clinical and psychological course of diabetes from adolescence to young adulthood: a longitudinal cohort study. Diabetes Care.

[6] Ibfelt EH, Wibaek R, Vistisen D (2022). Trajectory and predictors of Hba1C in children and adolescents with type 1 diabetes-a Danish nationwide cohort study. Pediatr Diabetes.

[7] Prahalad P, Yang J, Scheinker D (2019). Hemoglobin A1C trajectory in pediatric patients with newly diagnosed type 1 diabetes. Diabetes Technol Ther.

[8] Samuelsson U, Anderzen J, Åkesson K (2021). The importance of low Hba1C during childhood on glycaemic control in adulthood and the risk of late complications. Acta Paediatr.

[9] Samuelsson U, Anderzén J, Gudbjörnsdottir S (2016). Teenage girls with type 1 diabetes have poorer metabolic control than boys and face more complications in early adulthood. J Diabetes Complications.

[10] Arnqvist HJ, Westerlund MC, Fredrikson M (2022). Impact of Hba1C followed 32 years from diagnosis of type 1 diabetes on development of severe retinopathy and nephropathy: the VISS study. Diabetes Care.

[11] Dahlquist G, Blom L, Holmgren G (1985). The epidemiology of diabetes in Swedish children 0-14 years--a six-year prospective study. Diabetologia.

[12] Ostman J, Arnqvist H, Blohmé G (1986). Epidemiology of diabetes mellitus in Sweden. results of the first year of a prospective study in the population age group 15-34 years. Acta Med Scand.

[13] Gudbjörnsdottir S, Cederholm J, Nilsson PM (2003). The national diabetes register in Sweden: an implementation of the St. vincent declaration for quality improvement in diabetes care. Diabetes Care.

[14] Schön S, Ekberg H, Wikström B (2004). Renal replacement therapy in Sweden. Scand J Urol Nephrol.

[15] Wilkinson CP, Ferris FL, Klein RE (2003). Proposed International clinical diabetic retinopathy and diabetic macular edema disease severity scales. Ophthalmology.

[16] Arnqvist H, Wallensteen M, Jeppson JO (1997). [Standards for long-term measures of blood sugar are established]. Lakartidningen.

[17] Hoelzel W, Weykamp C, Jeppsson J-O (2004). IFCC reference system for measurement of hemoglobin A1C in human blood and the National standardization schemes in the United States, Japan, and Sweden: a method-comparison study. Clin Chem.

[18] Kullberg CE, Bergström A, Dinesen B (1996). Comparisons of studies on diabetic complications hampered by differences in GHb measurements. Diabetes Care.

[19] Treviño G (2007). Consensus statement on the worldwide standardization of the hemoglobin A1C measurement: the American diabetes association, European association for the study of diabetes, international federation of clinical chemistry and laboratory medicine, and the international diabetes federation: response to the consensus committee. Diabetes Care.

[20] Maddaloni E, Carlone A, Pitocco D (2023). Glycated haemoglobin in the first year after diagnosis of type 1 diabetes is an independent risk factor for diabetic retinopathy: the IMDIAB 25 years follow-up study. Diabetes Obes Metab.

[21] Nathan DM, Bayless M, Cleary P (2013). Diabetes control and complications trial/epidemiology of diabetes interventions and complications study at 30 years: advances and contributions. Diabetes.

[22] Zachrisson I, Brismar K, Hall K (1997). Determinants of growth in diabetic pubertal subjects. Diabetes Care.

[23] Barker A, Lauria A, Schloot N (2014). Age-dependent decline of Β-cell function in type 1 diabetes after diagnosis: a multi-centre longitudinal study. Diabetes Obes Metab.

[24] Schwandt A, Hermann JM, Rosenbauer J (2017). Longitudinal trajectories of metabolic control from childhood to young adulthood in type 1 diabetes from a large German/Austrian registry: a group-based modeling approach. Diabetes Care.

[25] Hoffman RP, Vicini P, Sivitz WI (2000). Pubertal adolescent male-female differences in insulin sensitivity and glucose effectiveness determined by the one compartment minimal model. Pediatr Res.

[26] Ludvigsson J, Carlsson A, Deli A (2013). Decline of C-peptide during the first year after diagnosis of type 1 diabetes in children and adolescents. Diabetes Res Clin Pract.

